# The T-box transcription factor, TBX3, is sufficient to promote melanoma formation and invasion

**DOI:** 10.1186/1476-4598-12-117

**Published:** 2013-10-07

**Authors:** Jade Peres, Sharon Prince

**Affiliations:** 1 Department of Human Biology, Faculty of Health Sciences, University of Cape Town, Observatory, Cape Town 7925, South Africa

**Keywords:** TBX3, Melanoma, Migration, Invasion

## Abstract

The T-box transcription factor, TBX3, is overexpressed in several cancers and has been proposed as a chemotherapeutic target. Several lines of evidence suggest that TBX3 may be a key contributor to malignant melanoma, a highly aggressive and intractable disease. Using *in vitro* and *in vivo* assays we demonstrate here for the first time that overexpressing TBX3 in non-tumourigenic early stage melanoma cells is sufficient to promote tumour formation and invasion. Furthermore, we show that TBX3 may play an important role as a reciprocal switch between substrate dependent cell proliferation and tumour invasion.

## Letter to the Editor

### Background

Malignant melanoma is a highly aggressive and intractable disease which has a rising incidence surpassing all other cancers [1]. It is therefore imperative to identify new targets for the development of improved therapies to treat melanoma. To this end, an understanding of the molecular mechanisms underpinning the stepwise progression of melanocytes to metastatic melanoma is important. Indeed, in the last decade, several transcription factors have been identified as potential therapeutic targets including MITF, PAX3, SNAIL and the developmentally important T-box transcription factor TBX3 [2-4].

TBX3 is overexpressed in a number of cancers including a subset of melanomas but its precise role in the progression of this disease still needs to be clarified. Earlier studies pointed to TBX3 contributing to the oncogenic process by functioning as an anti-senescence factor by directly repressing the negative cell cycle regulator p19/p14ARF [5,6]. More recent studies however suggest that TBX3 may function in advanced stages of melanoma. Hoek and colleagues demonstrated that TBX3 was upregulated in approximately 55% of cell lines obtained from advanced melanoma lesions [7]. Furthermore, we have shown that knocking down TBX3 in vertical growth phase (VGP) and metastatic melanoma cells inhibits cell migration and tumour formation [8] and TBX3 was demonstrated to repress the E-cadherin gene which is characteristically downregulated in VGP melanoma [9]. Furthermore, Boyd and co-workers demonstrated that oncogenic B-RAF is able to upregulate TBX3 expression which in turn represses E-cadherin levels [10].

## Findings

### WM1650-TBX3 cells have decreased proliferative ability

To determine if TBX3 is not only essential but sufficient to promote melanoma formation, we stably transfected the non-tumourigenic human WM1650 radial growth phase (RGP) melanoma cell line, which expresses undetectable levels of TBX3, with the pEGFP-N3 vector in which the cytomegalovirus promoter drives the expression of a green fluorescent protein tagged human TBX3.

**Figure 1:**
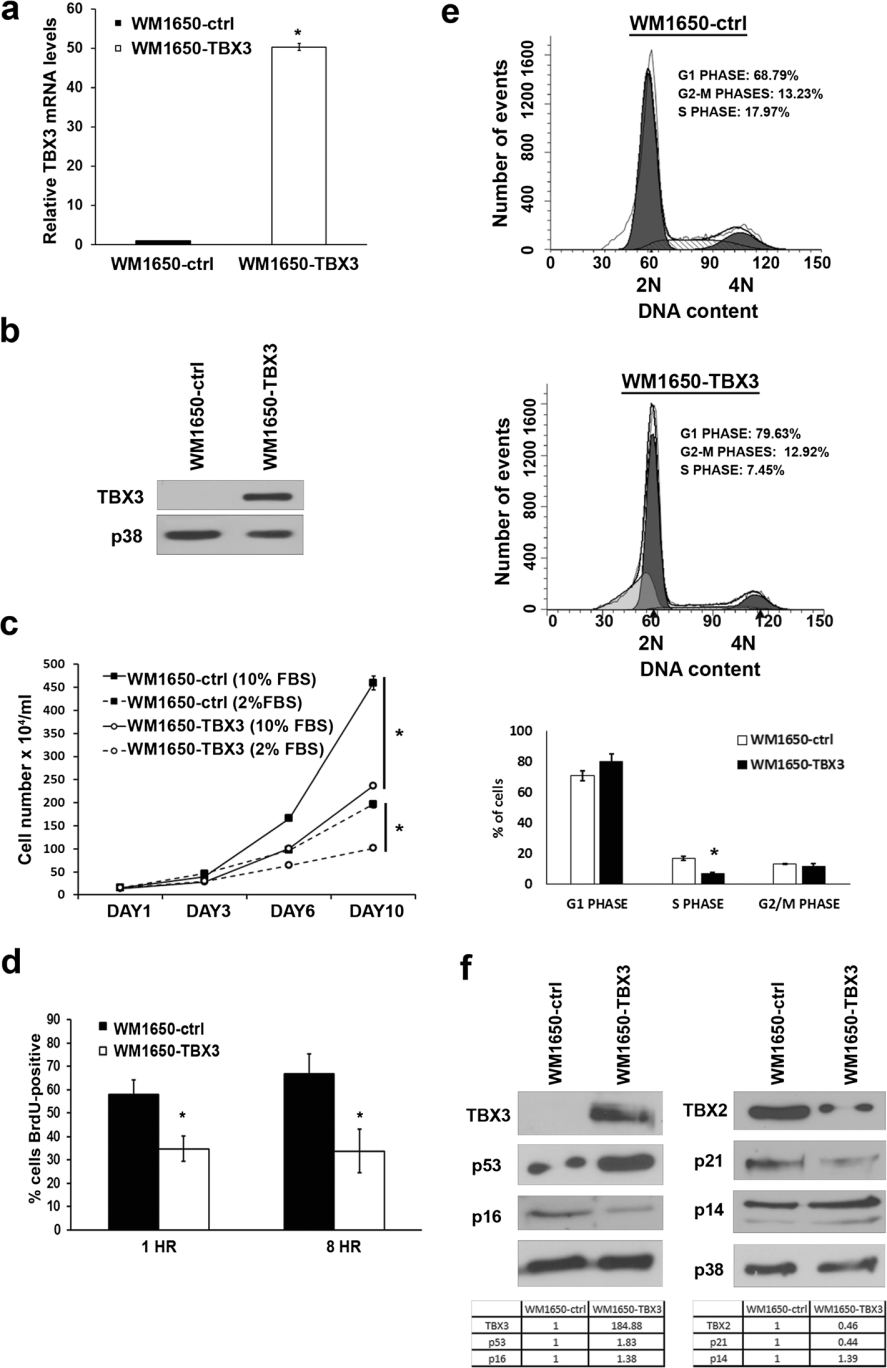
**WM1650-TBX3 cells have decreased proliferative ability. (a)** Real-time PCR (each data point represents the mean ± SD from at least three independent experiments (*p < 0.05)) and **(b)** western blot (representative of three different experiments) analyses of TBX3 mRNA and protein levels respectively in WM1650-ctrl and WM1650-TBX3 cells. p38 was used as a loading control. **(c)** Growth curve assays of cells grown in medium supplemented with 10% or 2% FBS. Each data point represents the mean ± SD from at least three independent experiments (*p < 0.05) **(d)** 5-bromo-2-deoxyuridine (BrdU) incorporation assay. Graph shows an average of BrdU-positive cells expressed as a percentage of total cells counted at 1 hr and 8 hrs in 20 fields of view (*p < 0.05, mean +/− SD). **(e)** Cell cycle distribution was determined by staining cells with propidium iodide and measuring their DNA content by flow cytometry. Percentage of cells in each phase of the cell cycle are shown in the graph below (*p < 0.05, mean +/− SD). **(f)** Western blotting (representative of three different experiments) of the indicated proteins in WM1650-ctrl and WM1650-TBX3 cells. The expression of TBX2, p53, p21, p16 and p14 was quantified as the densitometry value (pooled from three different experiments) analysed by UN-SCAN-IT gel 6.1 software and was normalised to p38 levels as shown in the tables below.

Western blot and qRT-PCR analyses confirmed that TBX3 was successfully overexpressed in the WM1650-TBX3 cell line established (Figure 1a, b).

We previously reported that VGP and metastatic melanoma cells in which TBX3 was depleted maintained a higher proliferative ability than their control cells [8]. It was therefore hypothesised that the overexpression of TBX3 in RGP cells would lead to a decrease in their proliferative ability.

Indeed growth curves and pulsing the cells with 5-bromo-2-deoxyuridine (BrdU), a synthetic thymidine analogue confirmed this to be the case (Figure 1c, d). To further explore the reason(s) for the decrease in cell proliferation, the cell cycle profile of WM1650-TBX3 and WM1650-ctrl cells were compared by flow cytometry. The results showed that there were 10% more WM1650-TBX3 cells in the G1 phase, but 10.5% fewer in the S-phase (Figure 1e). Furthermore, while the WM1650-TBX3 cells had reduced levels of p21, p16 and TBX2, they had increased levels of the tumour suppressor proteins, p53 and p14 (Figure 1f). This is significant because TBX2, a homologue of TBX3, was shown to be a powerful pro-proliferative factor in melanoma and breast cancer cell lines which express both TBX2 and TBX3 [8,11] and there has been an indication that TBX2 and TBX3 can repress each other [9]. Together these results show that TBX3 impacts negatively on melanoma cell proliferation.

### WM1650-TBX3 cells have increased anchorage independence and migratory ability

**Figure 2:**
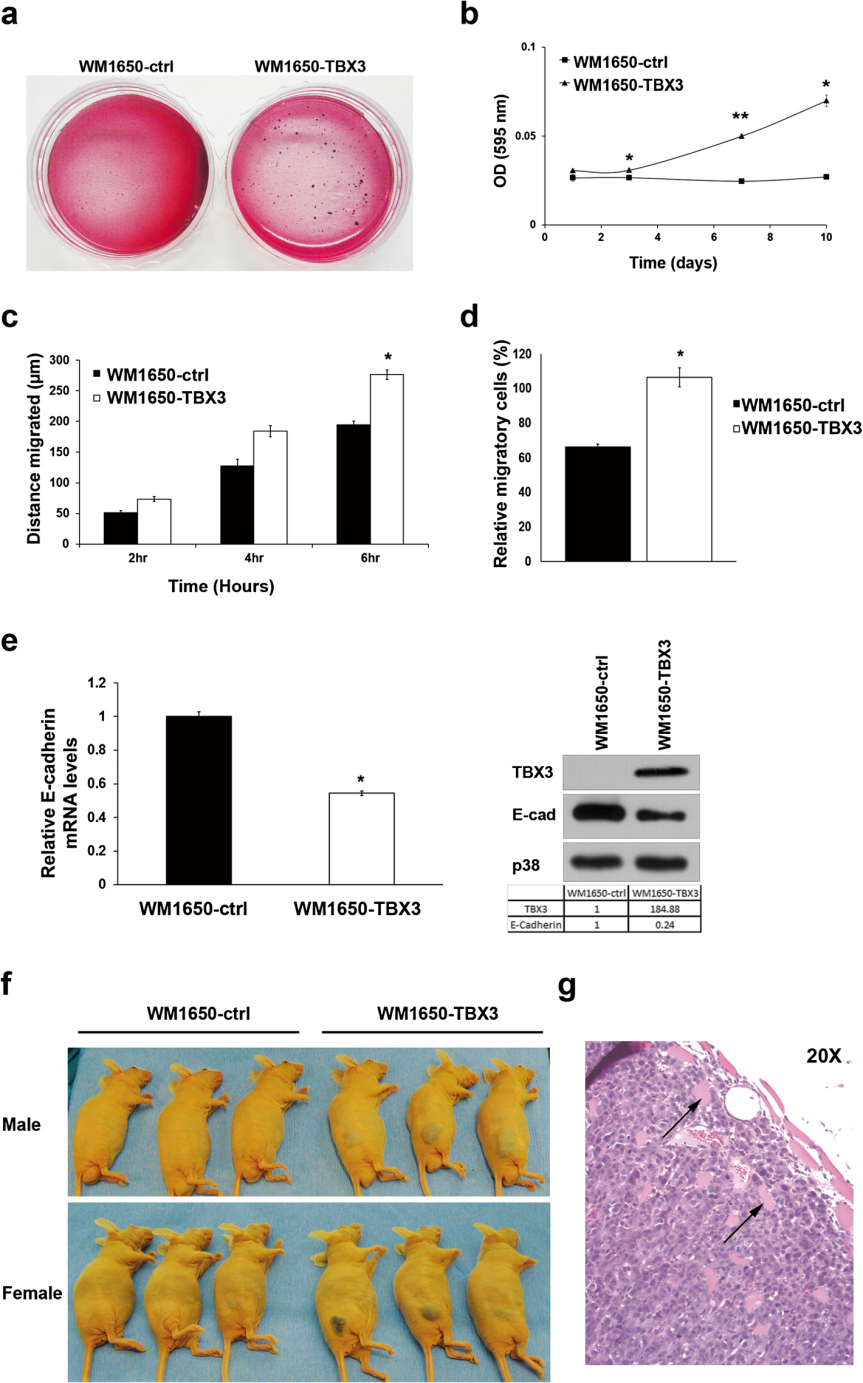
**WM1650-TBX3 cells have increased anchorage independence and migratory ability.** Cell growth **(a)** in a 1% agar slurry and visualised with p-iodonitrotetrazolium chloride stain and **(b)** in 0.8% methylcellulose and assessed by the MTT assay. Each data point represents the mean ± SD from at least three independent experiments (*p < 0.05; **p < 0.0009). **(c,d)** Migratory ability of the WM1650-TBX3 and WM1650-ctrl cells was compared using **(c) ***in vitro* scratch and **(d)** transwell motility assays. Each data point represents the mean ± SD from at least three independent experiments (*p < 0.05). **(e)** Real-time PCR analysis (left panel, each data point represents the mean ± SD from at least three independent experiments (*p < 0.05)) and western blotting (right panel; representative of three different experiments) of TBX3 and E-cadherin expression in WM1650-ctrl and WM1650-TBX3 cells. The expression of E-cadherin was quantified as the densitometry value (pooled from three different experiments) analysed by UN-SCAN-IT gel 6.1 software and was normalised to p38 levels as shown in the table below. **(f)** WM1650-TBX3 or WM1650-ctrl cells were injected subcutaneously into the flanks of 6 nude mice (3 males and 3 females for each cell line) and 29 days post-injection, mice were euthanized, photographed and tumours excised for histological analyses. **(g)** Representative tumour section stained with haematoxylin and eosin and photographed at 20× magnification. Black arrows show tumour cells invading skeletal muscle.

To further explore the possibility that TBX3 may drive RGP cells to a more aggressive melanoma phenotype the anchorage independent growth and colony forming ability of the WM1650-ctrl and WM1650-TBX3 cells were analysed using soft agar and methyl cellulose cell viability methylthiazol tetrazolium (MTT) assays. The results show that TBX3 promoted anchorage independent growth since the WM1650-TBX3 cells formed more and larger colonies (Figure 2a, b). Similarly, the two-dimensional scratch (Figure 2c) and transwell motility (Figure 2d) assays showed that the WM1650-TBX3 cells migrated significantly faster than the WM1650-ctrl cells. To confirm that the mechanism by which TBX3 affects melanoma cell adhesion and migration is through repressing E-cadherin, we investigated the levels of this cell adhesion molecule. Our results show that, compared to WM1650-ctrl cells, WM1650-TBX3 cells had lower levels of E-cadherin mRNA and protein (Figure 2e). To confirm the above findings *in vivo*, the tumour forming ability of WM1650-TBX3 and WM1650-ctrl cells was compared in nude mice. While 100% of mice injected with WM1650-TBX3 cells formed tumours ranging from 8 to 17.84 mm in diameter, 0% of mice injected with WM1650-ctrl cells formed tumours (Figure 2f). The ability of TBX3 to drive non-tumourigenic cells to form tumours is shown to be highly sensitive and specific (100% in both cases). H & E staining of tumour sections confirmed evidence of tumour cell infiltration into the skeletal muscle of the hind limb of mice injected with WM1650-TBX3 cells (Figure 2g black arrows).

## Conclusion

This study demonstrates for the first time that increased levels of TBX3 are sufficient to promote tumour formation and invasion of non-tumourigenic melanoma cells *in vivo*. We hypothesise that this occurs as a result of TBX3 repressing E-cadherin which leads to anchorage-independent growth and migration. Furthermore, we show that TBX3 may play an important role as a reciprocal switch between substrate dependent cell proliferation and tumour invasion.

## Abbreviations

VGP: Vertical growth phase; RGP: Radial growth phase; BrdU: 5-bromo-2-deoxyuridine; MTT: Methylthiazol tetrazolium.

## Competing interests

The authors declare that they have no competing interests.

## Authors’ contributions

SP conceived of the study, and supervised. JP conducted all assays. SP and JP contributed to the overall experimental design and wrote the manuscript together. Both authors read and approved the final manuscript.
